# Effect of Alginate-Microencapsulated Hydrogels on the Survival of *Lactobacillus rhamnosus* under Simulated Gastrointestinal Conditions

**DOI:** 10.3390/foods10091999

**Published:** 2021-08-26

**Authors:** Khyati Oberoi, Aysu Tolun, Zeynep Altintas, Somesh Sharma

**Affiliations:** 1 School of Bioengineering and Food Technology, Shoolini University, Solan 173229, India; khyatioberoi123@gmail.com; 2 Food Engineering, Ankara University, Ankara 06110, Turkey; atolun@ankara.edu.tr; 3 Institute of Chemistry, Technical University of Berlin, Straße des 17. Juni 124, 10623 Berlin, Germany

**Keywords:** *Lactobacillus rhamnosus*, emulsions, microencapsulation, alginate-microencapsulated hydrogels, alginate, xanthan gum, simulated gastrointestinal conditions

## Abstract

Thanks to the beneficial properties of probiotic bacteria, there exists an immense demand for their consumption in probiotic foods worldwide. Nevertheless, it is difficult to retain a high number of viable cells in probiotic food products during their storage and gastrointestinal transit. Microencapsulation of probiotic bacteria is an effective way of enhancing probiotic viability by limiting cell exposure to extreme conditions via the gastrointestinal tract before releasing them into the colon. This research aims to develop a new coating material system of microencapsulation to protect probiotic cells from adverse environmental conditions and improve their recovery rates. Hence, *Lactobacillus rhamnosus* was encapsulated with emulsion/internal gelation techniques in a calcium chloride solution. Alginate–probiotic microbeads were coated with xanthan gum, gum acacia, sodium caseinate, chitosan, starch, and carrageenan to produce various types of microcapsules. The alginate+xanthan microcapsules exhibited the highest encapsulation efficiency (95.13 ± 0.44%); they were simulated in gastric and intestinal juices at pH 3 during 1, 2, and 3 h incubations at 37 °C. The research findings showed a remarkable improvement in the survival rate of microencapsulated probiotics under simulated gastric conditions of up to 83.6 ± 0.89%. The morphology, size, and shape of the microcapsules were analyzed using a scanning electron microscope. For the protection of probiotic bacteria under simulated intestinal conditions; alginate microbeads coated with xanthan gum played an important role, and exhibited a survival rate of 87.3 ± 0.79%, which was around 38% higher than that of the free cells (49.4 ± 06%). Our research findings indicated that alginate+xanthan gum microcapsules have a significant potential to deliver large numbers of probiotic cells to the intestines, where cells can be released and colonized for the consumer’s benefit.

## 1. Introduction

The recent decades have witnessed an increased interest in functional food and nutrition, as well as their impact on human health. This has led companies to use them in commercial foods [1]. As functional compounds, probiotics have been used for the development of a wide range of functional food products [2], which has considerably increased the global consumption of probiotic foods in recent years. The world market of probiotics was estimated as USD 49.4 billion in 2018, and is projected to reach USD 69.3 billion by 2023 [3]. Probiotics are defined by the World Health Organization as ‘‘living microorganisms which upon ingestion in certain numbers, exert health benefits beyond inherent general nutrition” [4]. It has been recommended that in order to produce a therapeutic benefit, probiotic bacteria in food should be present at levels of least 10^6^ cfu g^−1^ or mL^−1^ (live microorganisms per g or mL) at the time of consumption [1]. When administered in adequate amounts, the probiotic organisms confer their health benefits through inhibition of pathogen growth by blocking the adhesion sites of pathogenic bacteria while maintaining health-promoting gut microflora [2,5]. These living bacteria have the capacity of controlling intestinal infection, regulating serum cholesterol levels, boosting the host’s immune system, and improving lactose utilization in persons who have suffered from lactose malabsorption. They also show a positive impact on suppressing colon cancer and irritable bowel syndrome [6,7].

It was found that some probiotic bacteria, such as *Lactobacillus johnsonii*, *L. rhamnosus,* and *Saccharomyces boulardii,* provide a healthy gut flora and contribute to the host’s health [2,8]. Thanks to its clinically proven health-promoting effects, *L. rhamnosus* is employed as the probiotic model, and it has shown a biofilm-forming ability in vitro [5]. To confer their health benefits, the probiotic cells must retain their viability during food processing and storage, as well as gastrointestinal transit through the acidic stomach and intestine [7,8]. However, there are several limitations for the use of probiotics in foods and beverages. Several factors are directly associated with their growth and stability rate, including pH, storage temperature, processing conditions, and environment of the digestive system. A low pH is one of the most important factors restricting the survival rate of probiotic bacteria [9]. Hydrogen ions ruin probiotic cells by disrupting mass transfer through the cell membrane. Fermentation, processing conditions, and storage temperature are the other factors that play an important role in probiotic stability in food products [1,2,10]. Temperatures above 45–50 °C lead to a reduction in free probiotic cell viability during food processing. Moreover, transition through the gastrointestinal tract is still a major challenge to obtaining the minimum suggested concentration of viable cells to provide the aforementioned benefits [9].

In order to improve their survival in such adverse conditions, encapsulation of probiotics in hydrocolloid beads is generally used to improve probiotics’ survival during digestion, considering the limitations of free probiotics’ survival in food processing and during gastrointestinal transit [2,8]. Microencapsulation is a technology of packing liquids, solids, and gaseous materials into tiny capsules that release those contents at controlled rates over long periods of time [11,12]. Probiotics can be encapsulated with this technique so that they can be released at a controlled rate under specific conditions [13,14]. It has been confirmed that encapsulated bacteria can survive better than free cells during gastric transit and harsh environmental conditions. Nevertheless, materials and methodologies, as well as the coating material used for their production, should be chosen carefully by preserving their vitality during the encapsulation process [15].

The major methods used for probiotic encapsulation are extrusion, emulsion, and spray-drying. These techniques each have their own unique and specific characteristics that suit the encapsulation of probiotics. Spray-drying presents a great flexibility, but the process temperature is an important drawback of this technique for the encapsulation of probiotics. Despite the fact that the extrusion method utilizes a huge diversity of machines and industrial components for generating capsules from different polymer mixtures, the utilization of this technique at a large scale requires in-depth studies and a significant amount of investments. Emulsification is clearly one of the most common encapsulation techniques for producing capsules smaller than 100 µm at the laboratory scale [2]. In this regard, some carrier matrixes have been used to cover the probiotic cells. Polysaccharides (e.g., alginate, carrageenan, and chitosan) are typically used, and effectively protect the cells from the acidic environment of the stomach and subsequently release the cells gradually into the suitable intestinal sections of the gut [5,16].

Considering the protection of probiotics against the harsh conditions of digestion, a wide variety and combinations of coating materials have been studied. Characteristics of the materials used play a key role because the viability of encapsulated probiotic cells depends on the physicochemical properties of the material. Several encapsulation agents, including polysaccharides derived from algae (k-carrageenan, alginate), plants (pectin and starch derivatives, gum arabic), or bacteria (gellan, xanthan) and animal proteins have been investigated for use in the probiotic microencapsulation method. In this regard, hydrocolloids are popular wall materials for the encapsulation of food ingredients. The type and concentration of the coating material, particle size, initial cell number, and bacterial strains are some other parameters to be considered [9]. Of note, particular attention also needs to be paid when choosing the right material as food grade and approved by regulatory authorities [2].

Among the encapsulating materials used, alginate is the most commonly employed polymer for immobilizing viable cells, due to its strong capacity to be cross-linked and the different mild gelling characteristics that change based on the molecular weight. The main reasons behind its common utilization in the microencapsulation of probiotics include its GRAS (generally regarded as safe) status worldwide as a food additive, its lack of toxicity, low cost, simplicity, and biocompatibility [2]. However, when it comes to protecting cells from low-pH environments, there is a drawback to using alginate due to the fact that alginate beads present very porous capsules. Namely, at very low pHs, cross-linked alginate matrix systems are reduced, and this reduction in alginate molecular weight causes a faster degradation and release of active ingredients [9]. Some works mention that alginate microbeads, without the application of a coating, have the capacity to protect probiotics during food storage, but not upon exposure to low-pH solutions, such as in gastrointestinal conditions [2]. Mixing alginate with other polymers, such as chitosan and starch, appears to be a solution for the enhancement of microcapsules’ resistance to acidic media [5,9].

Xanthan gum is another coating material that has been used in combination with gellan gum to improve the protection capacity of microcapsules for probiotic bacteria (*L. plantarum* and *L. rhamnosus*) [17]. The most relevant properties of xanthan gum used in probiotic microencapsulation are its ability to keep microparticles in suspension without greatly raising viscosity, its tolerance to enzyme degradation, and acid resistance [18]. Milk proteins, such as casein and whey protein, can also be used to encapsulate probiotics because of their excellent gelation properties. Considering the extreme conditions of the stomach, these proteins are able to create a higher local pH value within the protein matrix of the capsules, thanks to the buffering capacity of the proteins. Their amphoteric feature makes it possible to mix them with negatively charged polysaccharides such as alginate, carrageenan, or pectin [19].

In this study, we aimed to evaluate the effect of different coating materials (xanthan gum, gum acacia, sodium caseinate, chitosan, starch, carrageenan) on the efficiency and survivability of alginate-encapsulated *Lactobacillus rhamnosus* under different pH, temperature, NaCl, and simulated digestive system conditions. This is the first study in which such a variety of coating materials were investigated and compared for encapsulating probiotic bacteria. Moreover, the antimicrobial activity of the optimized microcapsules against some indicator microorganisms (*S. aureus, E. coli, B. cereus, S. typhimurium*) were also successfully evaluated. 

## 2. Materials and Methods

### 2.1. Materials and Chemicals

Sodium alginate with a medium viscosity and high mannuronic acid without inulin were purchased from Hi-media (Mumbai). They were prepared in distilled water and autoclaved at 121 °C for 15 min. The low-molecular-weight chitosan (deacetylated chitin, Hi-Media, Laboratories Mumbai), was prepared in distilled water and pure hydrochloric acid (Loba Chemie, Pvt Ltd.—Mumbai, India). Xanthan gum, gum acacia, and carrageenan were purchased from Loba Chemie, Pvt Ltd.—Mumbai, India. Starch, sodium caseinate, pepsin, trypsin, calcium chloride, sodium chloride, sodium bicarbonate, trisodium citrate, sodium hydroxide, potassium chloride, phosphate buffer saline (pH 7.2), and hydrochloric acid were purchased from Hi-media Laboratories (Mumbai, India). Glacial acetic acid with molar mass of 60.05 g mol^−1^ was bought from Merck (Darmstadt, Germany). Pancreatin, peptone, and bile salt were obtained from Loba Chemie, Pvt Ltd. (Mumbai, India). The De Man Rogosa and Sharpes (MRS) broth and MRS agar used in this work were purchased from Hi-media Laboratories (Mumbai). Indicator microorganisms used for the antimicrobial activity; i.e., *Escherichia coli* (MTCC No-432), *Staphylococcus aureus* (MTCC No-96), *and Bacillus cereus* (MTCC No-430), were procured from the Institute of Microbial Technology (IMTECH; Chandigarh, India).

### 2.2. Bacterial Strain and Culture Preparations

Microencapsulated and free cells of the bacterial strain *L. rhamnosus* isolated from indigenous fermented foods were grown in MRS broth and incubated at 37 °C in the absence of oxygen for 24–48 h. The cultures were harvested by centrifugation at 8000 rpm for 15 min at 4 °C, followed by two washes with the triple-distilled water. To achieve a suspension comprising approximately 10 log (cfu mL^−1^) cells, the pellet was resuspended in 0.1% (*w*/*v*) peptone solution. The cell suspensions were freshly prepared for each experiment and then dissolved in 1% (*w*/*v*) sterile saline solution. The cell counts were calculated by the pour plate method using appropriate 10-fold dilutions onto MRS agar after 48 h of incubation at 37 °C [20].

### 2.3. Microencapsulation Procedure of the Microorganisms

To produce the microcapsules, alginate beads were initially prepared as microbeads. These microbeads were then coated with a polymeric matrix such as xanthan gum, gum acacia, sodium caseinate, chitosan, starch, or carrageenan. For preparation of microbeads using the external gelation process, the sodium alginate was prepared (1%, *w*/*v*) by dissolving it in distilled water and stirring at 65 °C for 20 min. A 5 mL sample of the bacterial suspension containing a viable count of 10^7^–10^8^ cfu mL^−1^ was added to the previously prepared alginate solution (95 mL) and stirred at 65 °C for 20 min until obtaining a homogenous solution. The cross-linking solution (10%) was prepared by dissolving calcium chloride in distilled water. The alginate solution was drawn into a 3 mL syringe with a 26 G needle and dropped manually into the cross-linking medium for the formation of the alginate beads. The beads were then filtered using a strainer, rinsed with distilled water, and stored in the refrigerator until further use.

The microencapsulation of *Lactobacillus rhamnosus* was performed using the method defined by Vodnar et al. [21]. The different polymeric matrix solutions (i.e., xanthan gum solution, gum acacia solution, sodium caseinate solution, chitosan solution, starch solution, and carrageenan solution) were prepared in distilled water (2%, *w*/*v*) and autoclaved at 121 °C for 15 min. Each polymeric matrix solution was mixed with sodium alginate beads (1:1). The mixture was homogenized into a sunflower oil containing 0.2% (*w*/*v*) and emulsified for 5 min by stirring at 400 rpm. Afterwards, a solution of glacial acetic acid (900 µL) was dissolved in 10 mL of sunflower oil, and the mixture was stirred for 10 min. To stabilize the microencapsulated beads, the emulsion was lowered into the sterile solution of CaCl_2_ (2% *w*/*v*). The oil coating on the top layer was filtered with Whatman filter paper, and the microbeads were washed in 500 mL of distilled water. The microbeads were then stored in saline water with the pH adjusted to 4.0 at 4 °C in the refrigerator until further use. As a control, the free nonencapsulated cultured bacteria were also collected and stored using the same procedure [22].

### 2.4. Particle Sizes of Encapsulated Microbeads 

Dynamic light scattering (DLS) studies were performed to confirm the average particle size of the beads. The particle sizes of encapsulated beads were determined at 25 °C using a Malvern Zetasizer Nano ZS system (Malvern Instruments, Ltd., Malvern, UK). For this, 2 mg of encapsulated beads was dissolved in 10 mL of distilled water, and the suspension was then filtered and used for analysis. The measurements were performed in a computer-controlled particle-size analyzer to determine the particle-size distribution. Each sample was analyzed in triplicate.

### 2.5. Characterization of Microbead Morphology

The morphologies of the encapsulated microbeads were analyzed by field emission scanning electron microscopy (FESEM) (Nova Nano SEM, 450, FEI Company, Hillsboro, OR, USA). For this, the encapsulated beads were taped to the stub and coated for 1–2 min with an Au–Pd coating using a sputter coater and then used for analysis under the FESEM at an accelerating voltage potential of 20 kV.

### 2.6. Microencapsulation Yield

The viable cell count of *L. rhamnosus* was calculated according to a modified method [7]. To release the entrapped bacteria from the capsules, 1 g of microbeads was dissolved in 9 mL of sterile tri-sodium citrate solution (2%, *w*/*v*) and vortexed at room temperature for 5 min. The samples were serially diluted with 0.1% peptone (*w*/*v*) and dissolved in MRS agar. The plate was incubated in anaerobic conditions at 37 °C for 48 h.

The encapsulation yield (*EY*) was calculated using Equation (1):

(1) $$EY\;\left(\%\right)\;=\left(\frac{N}{No}\right)\times 100$$

where *N* is the number of the viable entrapped bacterial cells (cfu mL^−1^) released from the beads, and *N*_0_ is the number of the free viable bacterial cells (cfu mL^−1^) added to the biopolymer mixture during the preparation of microbeads.

### 2.7. Growth Profile of Microencapsulated Cells 

Free and microencapsulated microbial cells were inoculated in a triplicate sterile MRS broth. The cell density was calculated as optical density (OD) every 2 h over a 24 h period at 600 nm by employing a UV-spectrophotometer (UV 2450, Thermo Fisher Scientific, Germany). The controls were obtained using the ODs of the bacteria-free broth and empty microbeads.

### 2.8. Tolerance to Bile Salts and Acid 

The ability of isolated *L. rhamnosus* to sustain different bile salt concentrations was studied according to the method described by Succi et al. [23]. In sterile test tubes, 10^8^ cfu mL^−1^ of bacterial suspension was inoculated in 10 mL of bile solution at different concentrations of bile salt (0, 1, 1.5, and 2%). Sterile distilled water with no bile salt was kept as control. The samples were serially diluted and then plated on MRS agar plates. Afterwards, the plates were incubated aerobically at 37 °C and at different time intervals (2, 4, and 6 h), and the colony counts were recorded.

The survivability of microencapsulated *L. rhamnosus* was tested by adjusting sterile MRS broth to pH 1, 2, and 3 using 1 mol HCl. Sterile distilled water at pH 7.0 was used as control. All tubes were filled with 10 mL of sterile MRS broth. The different sets of tubes were inoculated with a bacterial suspension (10^8^ cfu mL^−1^) at each pH (1, 2, 3, and 7). The tubes were then incubated at 37 °C for 2, 4, and 6 h, respectively. At the time of incubation, 1 mL of culture was taken from each tube and serially diluted 10-fold with 0.85% normal saline solution and poured on the MRS agar plates. The plates were then incubated at 37 °C for 24–48 h to determine the residual viable count [24].

### 2.9. Growth of Encapsulated L. rhamnosus at Different Temperatures

One gram of the bacterial strain *L. rhamnosus* encapsulated in alginate+xanthan, as well as 1 mL of free cells with cell density of 10^8^ cfu mL^−1^, were added to 10 mL of preheated sterile water and inoculated in different tubes. Distilled water was used as the suspending medium. Further, the individual tubes were incubated overnight at various temperatures of 5, 15, 37, and 45 °C, respectively. The growth of the strain at the different temperatures was compared with the control tube, which was incubated at 37 °C for 24 h. After incubation, the sample tubes were cooled to room temperature, and viable cells were counted in triplicate on MRS agar [9].

### 2.10. Growth of Encapsulated L. rhamnosus at Different NaCl Concentrations 

The sterilized test tubes containing 10 mL MRS broth with different NaCl concentrations (0, 2, 3, 4, and 6%) were inoculated with the bacterial suspension (100 µL) with a viable cell count (log10^8^ cfu mL^−1^). All test tubes were incubated at 37 °C for 24 h, after which the viability of free and microencapsulated cells was recorded in triplicate on MRS agar [25].

### 2.11. Survivability of Microencapsulated Cells after Incubation in Simulated Gastric Juice 

The simulated gastric juice (SGJ), which consisted of 10 mg mL^−1^ of pepsin and 0.02 M phosphate buffer solution (PBS), was adjusted to pH 3.0 with 1 M HCl and sterilized by autoclaving at 121 °C for 15 min. Microencapsulated or free probiotic samples (0.5 g) were inoculated on tubes (4.5 mL, preheated to 37 °C) containing the filtered and sterilized simulated gastric juice (SGJ) and incubated at 37 °C. After incubation, the viable cell count was assessed using the surface plate count method at 0, 1, 2, and 3 h time intervals. The survival rate (%) of free and microencapsulated bacteria was calculated using Equation (2):

(2) $$Survival\;rate\;\left(\%\right)\;=\left(\frac{N}{No}\right)\times 100$$

where *N* is the number of viable cells (cfu g^−1^) after exposure to the simulated gastric juice conditions, and *N*_0_ is the number of viable cells (cfu g^−1^) before exposure to the simulated gastric juice conditions.

### 2.12. Survival of Microencapsulated Cells after Incubation in Simulated Intestinal Juice

The simulated intestinal juice (SIJ) was prepared according to the method described by Gbassi et al. [26]. A solution of 6.5 g L^−1^ NaCl, 0.835 g L^−1^ KCl, 0.22 g L^−1^ CaCl_2_, 1.386 g L^−1^ NaHCO_3_, and 3 g L^−1^ bile salt was adjusted to pH 7.5 and sterilized at 121 °C for 15 min before adding pancreatin in a final concentration of 10 g L^−1^. The SIJ was inoculated with 10% of the microcapsules and incubated at 37 °C. The viable cell count was determined at 0, 1, 2, and 3 h time intervals, as previously stated.

### 2.13. Antimicrobial Activity of Probiotic Isolate L. rhamnosus

The isolated *L. rhamnosus* was further investigated for its antimicrobial activity against food-borne pathogens; i.e., *Escherichia coli* (MTCC No-432), *Staphylococcus aureus* (MTCC No-96), and *Bacillus cereus* (MTCC No-430). A concentration of approximately 10^8^ cfu mL^−1^ of the indicator strain was added to 10 mL of MRS agar and poured over the plate containing the producer after 24 h of anaerobic incubation at 37 °C. The bacterial lawns for the zones of inhibition surrounding the producer colonies were tested. Positive inhibition was described as a 5 mm or greater clear zone surrounding the producer’s colonies.

### 2.14. Statistical Analysis

The data obtained in this research were expressed as mean ± SD of triplicates and were analyzed using one-way analysis of variance (ANOVA); 0.05 was chosen as the level of statistical meaning. The mean and standard deviation (SD) were calculated by subjecting the values to SPSS 16.020 statistical analyses when required using Microsoft Excel 2007. The graphs were created using GraphPad Prism.

## 3. Results and Discussion

### 3.1. Particle Size of Encapsulated Microbeads

In the present study, the effect of different encapsulation agents (xanthan gum, gum acacia, sodium caseinate, chitosan, starch, and carrageenan) on the size of microcapsules were evaluated. As shown in Table 1, the particle size significantly varied between 188.2 ± 4.98 and 1307.3 ± 126.7 µm according to the encapsulation agent used.

**Figure 1:**
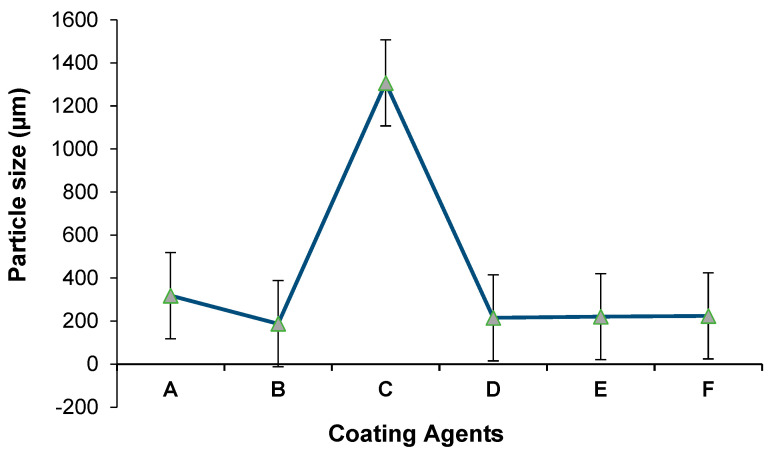
Particle-size distribution of the microcapsules: (**A**) alginate+xanthan gum; (**B**) alginate+gum acacia; (**C**) alginate+sodium caseinate; (**D**) alginate+starch; (**E**) alginate+chitosan; (**F**) alginate+carrageenan.

The particle-size distribution depending on the microencapsulation agent is presented in Figure 1. While the smallest size was obtained when using alginate+gum acacia, the largest microcapsule size was recorded in the case of using alginate+sodium caseinate as an encapsulation agent. The particle sizes prepared with alginate+sodium caseinate were around 1370.3 µm, and aggregation was observed in the sample. These aggregates could be attributed to the presence of casein in the encapsulation mixture, which consequently increased the particle size [27]. Of note, the composition, dispersion, and viscosity of the coacervate and supernatant phase influenced the size distribution, surface morphology, and internal porosity of the final microspheres [2].

Size and characteristics of the microcapsules are important parameters because these factors affect the water insolubility. Moreover, to maintain the structure of microcapsules during the passage through the gastrointestinal tract, they should be able to release probiotics in the intestinal tract. A large particle size affects the survivability of microencapsulated bacteria by producing large pores in hydrogels, allowing small molecules such as oxygen, acids, bile salts, or digestive enzymes to easily diffuse and inactivate the encapsulated bacteria [28]. Furthermore, an increasing bead diameter enhances the protective effect against extreme environmental factors [29]. However, oversized beads lead to inappropriate mouthfeel when consumed [30]. Earlier studies showed that capsules with a size larger than 100 µm should be avoided due to a “gritty” sensation, while an average size of 30 µm was not detected by sensorial analysis [2].

### 3.2. Particle Morphology

**Figure 2:**
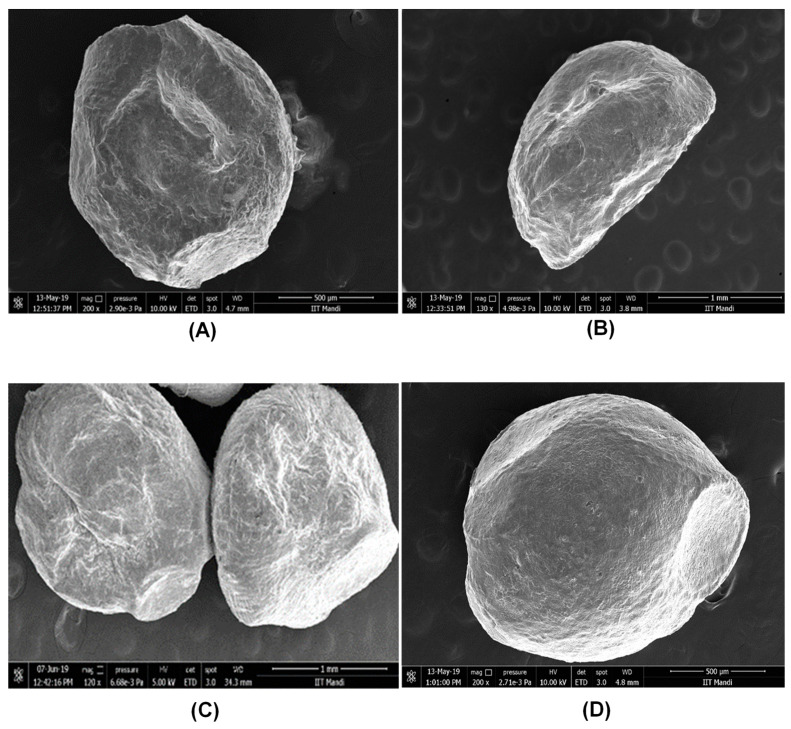

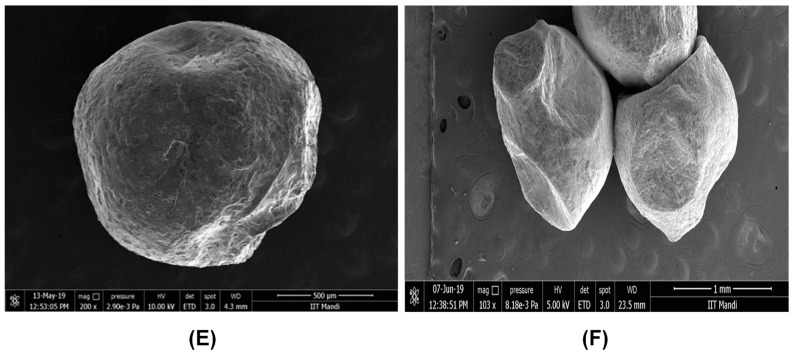
Field emission scanning electron microscopy images of (**A**) alginate+xanthan gum; (**B**) alginate+gum acacia; (**C**) alginate+carrageenan; (**D**) alginate+starch; (**E**) alginate+chitosan; and (**F**) alginate+sodium caseinate microcapsules.

Morphology of alginate microbeads coated with different encapsulation agents (xanthan gum, gum acacia, sodium caseinate, chitosan, starch, and carrageenan) were investigated. Figure 2 shows the FESEM images of the microcapsules loaded with *L. rhamnosus*. All the microbeads produced with different encapsulation agents showed significant differences in their shapes under the FESEM. Some microbeads were more spherical (Figure 2A,D,E) than those of others (Figure 2B,C,F). In parallel with our findings, the study conducted by Sultana et al. on a starch-coated alginate matrix loaded with probiotic bacteria showed that the microcapsules were generally spherical, but sometimes elliptical [31]. According to studies of sensation upon incorporating the microcapsules into the food, spherical and soft capsules produced a more pleasant sensation, while sharp or hard capsules revealed a rough and gritty sensation [2]. Moreover, more spherical microcapsules resulted in less surface area compared to rougher ones, meaning that spherical microcapsules present less surface area for contact with oxygen, which causes oxidation during storage [32]. Gandomi and coworkers evaluated the effect of microcapsules coated with alginate and chitosan on the viability of probiotic *L. rhamnosus* in apple juice, and they observed the coated beads as spherical. It was also noted that the sphericity played an important role in preventing cell overgrowth in encapsulated beads [7]. Spherical morphology was also obtained in another study in which chitosan-incorporated Ca–alginate beads were investigated [6].

### 3.3. Encapsulation Efficiency

The encapsulation efficiencies of various alginate beads, prepared in the presence of the previously mentioned six different encapsulating materials, were studied in detail, as shown in Table 1. The research findings showed that the microbeads loaded with *L. rhamnosus* had an encapsulation efficiency ranging between 70.06 ± 0.64 and 95.13 ± 0.44% based on the encapsulation material used. The use of the alginate+xanthan gum formulation exhibited the best efficiency (95.13 ± 0.44%) in comparison to other encapsulating agents. No significant difference was found between the efficiency of alginate+starch (93.80 ± 0.63%) and alginate+gum acacia (93.06 ± 0.74%) microbeads (*p* < 0.05). Both of these formulations also indicated a promising approach for loading probiotic bacteria. On the other hand, the alginate+chitosan formulation resulted in the lowest performance, with an encapsulation efficiency of 70.06 ± 0.64%. Overall, among the different encapsulation formulations we investigated, alginate+xanthan gum showed a significantly better outcome for loading probiotic bacteria in comparison to the other studies in the literature, in which whey protein+pullulan and alginate+psyllium+fenugreek were used as the encapsulation materials, respectively [27,30].

As a consequence of our findings, we concluded that alginate+xanthan gum was more durable, and increased the encapsulation efficiency of probiotic bacteria. Hence, alginate+xanthan gum was chosen for our further studies.

### 3.4. Growth Profile of L. rhamnosus in Microbeads

**Figure 3:**
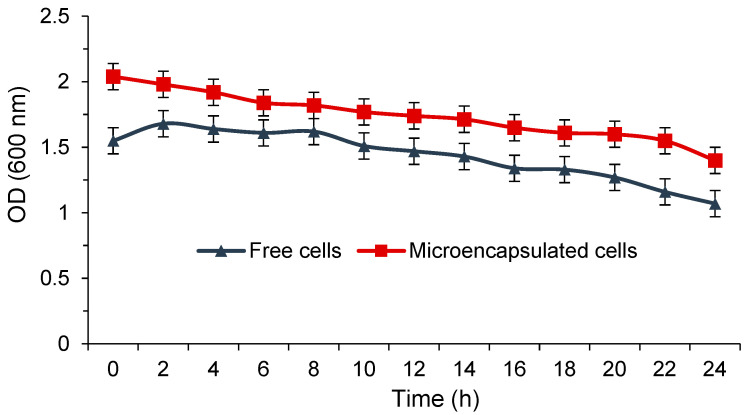
Cell density of free and microencapsulated *L. rhamnosus*.

The growth profile of *L. rhamnosus* in alginate+xanthan gum microbeads as compared to the free cells was investigated by measuring the cell density as a function of time. *L*. *rhamnosus* in microbeads reached a maximum cell density at about 2 h, as shown in Figure 3. The highest optical density of microencapsulated *L. rhamnosus* was found to be 1.98 times higher in 2 h than that of the free culture. However, when considering both conditions individually (i.e., free and microencapsulated cells), there were no significant differences in cell density during 24 h. A microenvironment with low shear stress was one of the possible reasons for a lack of a significant cell growth rate, which enhanced the microbial density of microbeads promoted by cell aggregation. To fit into the environmental conditions, the encapsulated cells required more time than the free ones. For microbial cell proliferation, the growth profiles proved that mass transfer and protection were provided by microbeads. In a previously reported study, the survival and cell density also were enhanced without any vast loss in cell count, and were accompanied by good probiotic capability [28].

### 3.5. Tolerance to Bile Salts and Acid 

The survival rate of microencapsulated *L. rhamnosus* confirmed an excellent tolerance to acid and bile environments without a significant loss in cell count while also offering a good probiotic viability. The reduction in the viable count of free cells was approximately 4–6 log cycles when exposed to different pH conditions in comparison to the control group (11.26 ± 0.15 log cfu mL^−1^ ), which was kept in sterile distilled water with no bile solution, pH 7) for 4 h, as shown in Table 2. The survival rate of the microencapsulated cells with alginate+xanthan gum at 2% bile concentration was 11.0 ± 0.04 log cfu mL^−1^, while it was only 8.97±0.02 log cfu mL^−1^ for free cells at 2 h. Furthermore, the survival decreased proportionally by the time the cells were subjected to bile salt solutions in this experiment, which was in good agreement with the literature [20,33], in which calcium alginate and alginate+chitosan were used as the encapsulation materials, respectively.

Argyri et al. demonstrated that 2.0% bile salt resulted in a maximum survival rate when added to the growth media [34]. The current study showed that the microencapsulated microbeads trapped with alginate+xanthan gum compared to free cells resulted in organic acid production and a pH decline. At pH 3.0, the microencapsulated cells survived with 10.2 ± 0.01 log cfu mL^−1^, and there was a 1–2 log reduction in cfu mL^−1^ as compared to pH 2 and pH 1 for 6 h, as depicted in Table 3. Although the pH value (3) used in this study for the selection of potential probiotic strains is a common pH value in the human stomach, it ensured the isolation of acid-tolerant strains [35]. Furthermore, there were no significant differences (*p* < 0.05) of the cell viability between different incubation times. These findings were in parallel with a previous study [9]. The researchers also concluded that the encapsulated *L. rhamnosus* effectively showed higher viability at different pH values compared to the nonencapsulated group. As per the earlier reports for pH 1.2, the nonencapsulated *L. acidophilus* was completely destroyed after 1 h of incubation, while the encapsulated *L. acidophilus* maintained above 10^6^ log cfu mL^−1^ at pH 1.5 after 2 h [36].

### 3.6. Growth at Different Temperatures

The effect of encapsulation treatments on the viability of *L. rhamnosus* during heat processing at 5, 15, 37, and 45 °C was evaluated for 24 h. Our research findings revealed that alginate microbeads coated with xanthan gum remarkably improved *L. rhamnosus’* stability against heat treatment. *L. rhamnosus* loaded in alginate+xanthan gum showed higher heat stability than the free cells. Nonencapsulated free *L. rhamnosus* cells, exposed to 5, 15, 37, and 45 °C, showed survival rates of 13.13 ± 1.79%, 25.93 ± 1.82%, 60.26 ± 0.23%, and 70.05 ± 0.87%, respectively (Table 4). On the other hand, the survival rates of encapsulated bacteria were found to be 84.73 ± 1.04%, 89.17 ± 1.17%, 92.7 ± 1.86%, and 96.86 ± 1.07% at the same temperatures, respectively. As can be seen in Table 4, even 45 °C was not lethal to the encapsulated *L. rhamnosus,* and the encapsulated cells still showed a significantly higher survival rate in comparison to free cells. Similarly, a 90% survival was reported earlier for encapsulated *L. plantarum* when the researchers subjected the microcapsules to a 50 °C heat treatment for 20 min [37]. Nevertheless, the current work achieved a far more desirable outcome when the timeframe of heat treatment was taken into consideration (24 h). Another study concluded that the encapsulated bacteria survived significantly (*p* < 0.05) better than the free cells during heat exposure to 55, 60, and 65 °C [9]. After exposure to heat treatment at 65 °C for 30 min, 40 g L^−1^ alginate + 10 g L^−1^ chitosan-encapsulated *L. rhamnosus* was decreased by only 2.55 log cycles, whereas free cells were reduced by about 5 log cycles [9].

### 3.7. Growth at Different NaCl Concentrations

The effects of encapsulation treatment on the viability of *L. rhamnosus* at different NaCl concentrations were studied, and the results are provided in Table 5. Accordingly, at various NaCl concentrations (0, 2, 3, 4, and 6%), the microencapsulated cells were able to grow at the end of 24 h at 37 °C. The survival rate of the microencapsulated cells in a 6% NaCl concentration was found to be 10.1 ± 0.05 log cfu mL^−1^, whereas the survival rate of free cells was recorded as 9.20 ± 0.05 log cfu mL^−1^. There was a statistically significant difference between the free cells and the encapsulated cells for all NaCl concentrations after 24 h. However, there was no statistically significant difference between different NaCl concentrations with respect to free cells and encapsulated cells individually. The growth (log cfu mL^−1^) observed for both free and microencapsulated cells at 0, 2, 3, 4, and 6% NaCl concentrations were (i) 9.29 ± 0.03, 9.25 ± 0.04, 9.23 ± 0.01, 9.21 ± 0.01, 9.20 ± 0.05; and (ii) 10.4 ± 0.05, 10.3 ± 0.01, 10.2 ± 0.05, 10.15 ± 0.05, and 10.1 ± 0.05 log cfu mL^−1^, respectively (Table 5). Of note, the microencapsulated cells showed more resistance at higher concentrations of NaCl than those of free cells. Therefore, it was evident that microencapsulation protected the cells at different salt concentrations, and hence effectively improved the efficacy of the encapsulated *L. rhamnosus.* Many publications have discussed the detrimental impact of sodium chloride on the activity of *Lactobacillus* species. Sodium chloride had a positive impact on the synthesis of bacteriocins such as sakacin P and lactacin 481, which are produced by *Lactobacillus sakei* and *Lactococcus lactis* [38]. However, when more than 7% NaCl was added, the cells expanded more slowly, and biomass production became less efficient due to the inactivation in generation time of microorganisms in the growth medium [39].

### 3.8. Viability in Gastric Juice

To improve the viability of *L. rhamnosus* during the exposure to the simulated gastric juice (SGJ), the survival rates of free and microencapsulated cells were investigated every hour during a total time period of 3 h. Overall results for the survival rate of free cells and probiotic bacteria encapsulated with alginate+xanthan are shown in Table 6. Irrespective of the measurement time, the encapsulated bacteria showed a significantly higher survival rate than that of the free cells in SGJ. This may be attributed to the fact that xanthan gum reduced the pore size of alginate and formed a double-layer membrane. As a result, the limited diffusion of gastric juice into the beads protected the encapsulated cells from interacting with the gastric juice [40]. Importantly, in terms of control samples, the survival rate of viable cells dramatically dropped within 3 h, from 54.8 ± 1.82 to 35.3 ± 0.94%, due to the acidic effect of the gastric juice. On the other hand, the microencapsulated cells maintained survival rates of 76.6 ± 0.75%, 74.6 ± 0.6%, and 68.0 ± 0.91% at the end of 1, 2, and 3 h, respectively. The viable count of the encapsulated *L. rhamnosus* was reduced from the initial count (10^8^ cfu mL^−1^) after exposure to SGJ for 3 h. The survival rate of *L. rhamnosus* with alginate+xanthan gum microencapsulated beads increased to 10.14 log cfu mL^−1^ as compared to free cells (9.92 log cfu mL^−1^). This showed that the alginate+xanthan gum encapsulating agent enhanced the survivability of the encapsulated microbeads under simulated gastric conditions.

One of the main objectives behind microencapsulation is to protect probiotic cells in the gastric environment during low-pH exposure [30]. To address this goal in human gastrointestinal disorders, probiotics should pass the small intestine and colonize the host at an adequate amount of 10^6^–10^7^ cfu g^−1^. In the present study, alginate+xanthan microcapsules increased the survival of cells in this environment, which was similar to the real gastric condition. The research findings proved that alginate+xanthan gum was the most effective encapsulating agent for the survival of *L. rhamnosus* under simulated gastric conditions. Xanthan gum is highly compatible with most thickener ingredients, including derived cellulose, starch, pectin, gelatin, dextrin, alginate, and carrageenan. In contrast to other trade polysaccharides, xanthan gum has a well-defined yield value, which affects emulsion stabilization and suspension media [41]. 

### 3.9. Viability in Intestinal Juice

In order to determine the tolerance of the free as well as encapsulated strains to the acidic pH, an in vitro gastric system was utilized. The viabilities of the microencapsulated cells in the simulated intestinal juice were reported after 0, 1, 2, and 3 h incubation times. According to the survival rate (%) results given in Table 6, the encapsulated probiotic cells exhibited significantly higher viabilities during the exposure time in comparison to the free cells. While the survival rate of viable cells in the control samples decreased in 3 h from 49.4 ± 0.6 to 28.7 ± 1.51%, the microencapsulated cells could maintain their survivability rate up to 82.7 ± 0.93% at the end of the first hour, and 78.2 ± 0.92 and 64.8 ± 0.93% after 2 and 3 h, respectively. Hence, the current findings confirmed that encapsulation certainly improved the survivability of *L. rhamnosus* in comparison to free cells in an in vitro gastric system.

Similar results were observed in simulated intestinal juice conditions, in which the viable cell count was reduced from the initial count (10^8^ cfu mL^−1^) by 9.8 log cycles, and when cells were entrapped in alginate+xanthan gum, the count was increased by 10.17 log cycles at 2 h. Although the viability of encapsulated *L. rhamnosus* was reduced during SGJ and SIJ treatments, the observed decreases were much lower compared to those of free cells. Furthermore, the survival rate of *L. rhamnosus* bacteria was better in microencapsulated cells as compared to the free cells in both conditions; i.e., SGJ and SIJ. This may be due to the fact that alginate can be stable in low-pH solutions, but swell in weakly basic conditions [7]. Based on our results, alginate microspheres can be used to protect *L. rhamnosus* from the acidity of gastric juice due to alginate’s stability in low-pH solutions. However, using alginate alone is not sufficient for intestinal system stability.

### 3.10. Antimicrobial Activity of L. rhamnosus against Indicator Microorganisms

The antimicrobial activity of *L. rhamnosus* against the indicator microorganisms such as *E. coli, S. aureus, B. cereus,* and *S. typhimurium* was studied. The results of our inhibition tests showed that the free and microencapsulated cells could form an inhibition zone on the solid medium. The zone of inhibition was found to be larger in microencapsulated beads as compared to free cells. As shown in Table 7, the zone of inhibition ranged between 15.4 ± 1.5 and 18.4 ± 2.5 mm and 18.4 ± 3.2 and 22.4 ± 1.5 mm for free and microencapsulated cells, respectively. The inhibition activity of *Lactobacillus* sp. could be due to the production of antimicrobial agents such as organic acids, hydrogen peroxide, and bacteriocin. *L. rhamnosus* that was entrapped in alginate+xanthan gum exhibited effective inhibition against the test organism. Furthermore, we concluded that there was no screening effect of microencapsulation on antimicrobial activity because microencapsulation does not control the functionality and metabolic action of trapped *L. rhamnosus* cells. Several research groups have also reported on their studies of the antimicrobial activity of *L. rhamnosus* against several pathogens, the results of which are in agreement with the current study [42,43,44]. As per these earlier studies, it was found that *Lactobacillus* and *Bifidobacterium* spp. also exerted an antimicrobial activity against the test organisms [45].

## 4. Conclusions

In this study, a microencapsulated probiotic (*L. rhamnosus*) was broadly evaluated in terms of cell viability against the harsh conditions of the gastrointestinal tract. The results proved that the application of probiotic microencapsulation is possible through a modified emulsion process. The developed microcapsules showed a suitable size and morphological structure for microbial cell growth and survivability. To produce alginate–probiotic microcapsules, among different biomaterials (xanthan gum, gum acacia, sodium caseinate, chitosan, starch, and carrageenan), the alginate + xanthan gum formulation showed a significantly higher encapsulation efficiency (95%) compared to other coating agents investigated in this work, as well as in the literature. The encapsulation of *L. rhamnosus* in alginate beads coated with xanthan gum increased the survival rate of the cells in stress conditions during gastrointestinal transition compared to the free bacteria. In addition, this technique enhanced the tolerance of bacteria to heat treatment applied at different temperatures while also allowing them to be metabolically active in appropriate conditions. The present investigations provide an innovative and new technique for protecting the probiotic culture of *L. rhamnosus* during the gastrointestinal transition. Considering the promising results achieved, the developed technique can be an attractive approach for in vivo studies to analyze the efficacy of the encapsulated materials. The current study also holds a potential application in different probiotic fruit-based juices with high acidities in the form of probiotic microcapsules. In this regard, the sensory evaluation of foods, including microencapsulated probiotic bacteria, will be the future perspectives from which to determine consumer responses in terms of sensory characteristics such as color, flavor, or taste.

## Figures and Tables

**Table 1:** Encapsulation efficiencies and particle sizes of different *L. rhamnosus* microcapsules.

Encapsulation Agent	Encapsulation efficiency		Particle Size	
	(%)		(µm)	
Alginate+Xanthan Gum	95.13 ± 0.44	a	318.40 ± 10.30	b
Alginate+Gum Acacia	93.06 ± 0.74	b	188.20 ± 4.98	f
Alginate+Sodium Caseinate	88.40 ± 0.63	c	1307.30 ± 126.70	a
Alginate+Chitosan	70.06 ± 0.64	d	215.20 ± 1.87	e
Alginate+Starch	93.80 ± 0.63	b	221.10 ± 3.89	d
Alginate+Carrageenan	77.90 ± 1.04	e	224.30 ± 6.88	c

a, b, c, d, e, f means within a row differed significantly (*p* < 0.05) (*n* = 3).

**Table 2:** Effect of bile salt on the viability of free and microencapsulated (alginate+xanthan gum) *L. rhamnosus* microcapsules (log cfu mL^−1^).

		Bile (%)							
	Time	1		1.5		2		0	
	(h)								
Free cells	2	6.84 ± 0.02	Aa	9.27 ± 0.01	Bg	8.97 ± 0.02	Cm	11.33 ± 0.05	Ds
	4	6.90 ± 0.02	Ac	9.26 ± 0.03	Bi	8.91 ± 0.02	Co	11.26 ± 0.15	Du
	6	6.97 ± 0.03	Ae	9.25 ± 0.01	Bk	8.84 ± 0.03	Cq	11.30 ± 0.10	Dw
MC cells	2	8.97 ± 0.02	Ab	10.28 ± 0.01	Bh	11.00 ± 0.04	Cn	12.35 ± 0.20	Dt
	4	7.94 ± 0.04	Ad	10.20 ± 0.01	Bj	9.94 ± 0.04	Cp	12.28 ± 0.04	Dv
	6	7.92 ± 0.04	Af	9.91 ± 0.05	Bl	9.87 ± 0.05	Cr	12.18 ± 0.08	Dx

Values are mean ± SD of three independent determinations (*n* = 3) of each isolate. A, B, C, D means within a row differed significantly (*p* < 0.05). a, b, c, d, e, f, g, h, i, j, k, l, m, n, o, p, q, r, s, t, u, v, w, x means within a column differed significantly (*p* < 0.05) (*n* = 3).

**Table 3:** Effect of pH on the viability of free and microencapsulated (alginate+xanthan gum) *L. rhamnosus* microcapsules (log cfu mL^−1^).

		pH							
	Time	1		2		3		7	
	(h)								
Free cells	2	6.97 ± 0.01	Aa	9.030 ± 0.01	Bg	10.40 ± 0.05	Cm	10.70 ± 0.67	Ds
	4	6.96 ± 0.01	Ac	9.050 ± 0.03	Bi	10.30 ± 0.15	Co	10.40 ± 0.71	Dr
	6	6.92 ± 0.01	Ae	7.970 ± 0.02	Bk	10.10 ± 0.11	Cq	10.10 ± 0.10	Dt
MC cells	2	8.72 ± 0.59	Ab	9.060 ± 0.04	Bh	10.10 ± 0.10	Cn	9.23 ± 0.01	Dq
	4	9.04 ± 0.07	Ad	9.030 ± 0.03	Bj	10.20 ± 0.02	Cp	9.23 ± 0.04	Ds
	6	8.96 ± 0.02	Af	9.270 ± 0.01	Bl	10.20 ± 0.01	Cr	9.40 ± 0.56	Du

Values are mean ± SD of three independent determination (*n* = 3) of each isolate. A, B, C, D means within a row differed significantly (*p* < 0.05). a, b, c, d, e, f, g, h, i, j, k, l, m, n, o, p, q, r, s, t, u means within a column differed significantly (*p* < 0.05) (*n* = 3).

**Table 4:** Effect of temperature on the survival rate of free and microencapsulated (alginate+xanthan gum) *L. rhamnosus* microcapsules.

Temperature(°C)	Survival Rate (%)			
	Free Cells		Microencapsulated Cells	
5	13.13 ± 1.79	Aa	84.73 ± 1.04	Be
15	25.93 ± 1.82	Ab	89.17 ± 1.17	Bf
37	60.26 ± 0.23	Ac	92.70 ± 1.86	Bg
45	70.05 ± 0.87	Ad	96.86 ± 1.07	Bh

A, B means within a row differed significantly (*p* < 0.05) (*n* = 3). a, b, c, d, e, f, g, h means within a column differed significantly (*p* < 0.05) (*n* = 3).

**Table 5:** Effect of NaCl concentrations on the viability of free and encapsulated (alginate+xanthan gum) *L. rhamnosus* microcapsules (log cfu mL^−1^).

NaCl	Free Cells		Microencapsulated Cells	
Concentration				
(%)				
0	9.29 ± 0.03	Aa	10.40 ± 0.05	Be
2	9.25 ± 0.04	Ab	10.30 ± 0.01	Bf
3	9.23 ± 0.01	Ac	10.20 ± 0.05	Bg
4	9.21 ± 0.01	Ad	10.15 ± 0.05	Bf
6	9.20 ± 0.05	Ad	10.10 ± 0.05	Bg

A, B means within a row differed significantly (*p* < 0.05) (*n* = 3). a, b, c, d, e, f, g means within a column differed significantly (*p* < 0.05) (*n* = 3).

**Table 6:** Effects of SGJ and SIJ on the survival rate of free and microencapsulated (alginate+xanthan gum) *L. rhamnosus* microcapsules.

		Survival Rate (%)			
Simulated	Time	Free Cells		Microencapsulated Cells	
Condition	(h)				
SGJ	0	54.80 ± 1.82	Ab	83.60 ± 0.89	Bi
	1	48.50 ± 0.78	Ad	76.60 ± 0.75	Bl
	2	44.10 ± 0.60	Ae	74.60 ± 0.60	Bn
	3	35.30 ± 0.94	Ag	68.00 ± 0.91	Bp
SIJ	0	49.40 ± 0.60	Aa	87.30 ± 0.79	Bh
	1	45.60 ± 0.94	Ac	82.70 ± 0.93	Bk
	2	34.10 ± 1.53	Ae	78.20 ± 0.92	Bm
	3	28.70 ± 1.51	Af	64.80 ± 0.93	Bo

A, B means within a row differed significantly (*p* < 0.05) (*n* = 3). Identical lowercase indicates no significant difference (*p* > 0.05). a, b, c, d, e, f, g, h, i, k, l, m, n, o, p means with in a column differed significantly (*p* < 0.05) (*n* = 3).

**Table 7:** Antimicrobial activity of the free cells and microencapsulated cells (alginate+xanthan gum) of *L. rhamnosus* microcapsules.

	Zone of Inhibition (mm)			
Test Microorganism	Free Cells		Microencapsulated Cells	
*E. coli*	17.7 ± 3.5	a	22.4 ± 1.5	b
*S.aureus*	15.4 ± 1.5	a	21.3 ± 3.0	b
*B. cereus*	18.4 ± 2.5	a	19.5 ± 2.5	b
*S. typhimurium*	16.7 ± 2.1	a	18.4 ± 3.2	b

a, b means within a row differed significantly (*p* < 0.05) (*n* = 3).

## Data Availability

The data presented in this study are available upon request from the corresponding authors.
